# Draft Genome Sequences of Fungi Isolated from Mars 2020 Spacecraft Assembly Facilities

**DOI:** 10.1128/mra.00464-22

**Published:** 2022-10-06

**Authors:** Atul M. Chander, Nitin K. Singh, Anna C. Simpson, Arman Seuylemezian, Christopher E. Mason, Kasthuri Venkateswaran

**Affiliations:** a Biotechnology and Planetary Protection Group, NASA-Jet Propulsion Laboratory, California Institute of Technology, Pasadena, California, USA; b Tri-Institutional Computational Biology & Medicine Program, Weill Cornell Medicine, New York, New York, USA; c WorldQuant Initiative for Quantitative Prediction, Weill Cornell Medicine, New York, New York, USA; University of California, Riverside

## Abstract

During the Mars 2020 mission, several fungal strains were isolated from surfaces where spacecraft components were assembled. Draft genome sequencing and characterization will help identify the genes responsible for radiation resistance, supporting the development of countermeasures to prevent fungal contamination of extraterrestrial environments.

## ANNOUNCEMENT

NASA planetary protection (PP) assays traditionally focus on the detection of endospore-forming bacteria (1, 2) because these can potentially survive on spacecraft launched to other planets. However, fungi and their conidia are other likely candidates for enduring spaceflight and other extreme space environmental conditions (3, 4). Identification and characterization of fungal isolates associated with spacecraft environments are essential to developing countermeasures for preventing fungal contamination on future exoplanet missions. As part of this effort, during microbial monitoring of the Mars 2020 cleanrooms, 26 fungal strains were isolated, and whole-genome sequencing (WGS) was carried out.

Sample collection, fungal isolation, and identification steps were performed as previously described (5). Briefly, samples were collected from cleanroom surfaces using moist polyester wipes and were agitated in sterile phosphate-buffered saline (PBS). The resulting PBS suspension was concentrated using an InnovaPrep CP150 concentrating pipette and spread onto plates containing different media (dichloran rose bengal agar, potato dextrose agar [PDA], and dichloran glycerol agar). Plates were incubated at 25°C for 7 to 14 days, and fungal strains were isolated, purified, and stored at −80°C for further identification. Under the same growth conditions, fungal isolates were revived on PDA plates to obtain pure culture and produce biomass for DNA isolation followed by internal transcribed spacer (ITS) region gene sequencing. The PDA plate-grown culture was scraped, and genomic DNA extraction was performed using the ZymoBIOMICS MagBead DNA kit (catalog no. D4302; Zymo Research, Irvine, CA). The DNA WGS libraries were prepared with an Illumina Nextera DNA Flex library preparation kit (6) and sequenced on the NovaSeq 6000 paired-end 2 × 150-bp platform using an S4 flow cell. FastQC v.0.11.7 and Fastp v.0.20.0 were used to filter and trim the reads, and the genomes were assembled using SPAdes v.3.11.1 (7, 8).

Amplicons of the ITS region were amplified from the DNA for all strains and sequenced, except for the strain FJI-L2-BK-P3 (5). Genus-level identification was made via BLAST searches against the NCBI ITS database (9). Subsequently, four additional secondary marker genes (*GAPDH*, *CaM*, *Tef1a*, and *BenA*) were extracted from WGS for species-level identification (10). Species-level identification was performed using a specific secondary marker gene suitable for each genus type as per recent recommendations and literature (Table 1) (10–15). The extracted marker genes were BLAST searched against NCBI nucleotide database (sequences from type material filter applied) to obtain the best percentage of identity matches, based on which the species names were assigned. Marker gene-based analysis of the 26 fungal genomes revealed the presence of nine genera and 13 fungal species. The majority of the species identified belonged to the genera *Penicillium* (4 species; 8 strains) and Aspergillus (2 species; 7 strains); the other seven genera each had a single species representative.

**TABLE 1:** Accession numbers, sampling locations, and assembly details for fungal strains isolated from the NASA spacecraft assembly facilities

Sample/strain name	Fungal species	Secondary marker gene used for identification^*a*^	% identity with secondary marker gene	Accession no.			Medium used	Facility^*b*^	No. of contigs	Genome size (bp)	*N*_50_ (bp)	Depth of coverage	G+C content (%)	No. of filtered reads
				ITS sequence	WGS	SRA								
FKII-L2-CM-DRAB1	Alternaria alternata	*GAPDH*	100.0	MT704920	JAKLNJ000000000	SRR18739383	Dichloran rose bengal agar	KSC-PHSF	137	34,215,686	1,397,744	115	51.05	2,054,427
FKII-L8-BK-P2B	Alternaria alternata	*GAPDH*	100.0	MT704925	JAKLNO000000000	SRR18739377	Potato dextrose agar	KSC-PHSF	105	33,359,331	1,380,845	55	51.3	2,650,675
FKI-L7-BK-DRAB2	Aspergillus calidoustus	*CaM*	99.8	MT704900	JAKLMU000000000	SRR18739397	Dichloran rose bengal agar	KSC-PHSF	619	61,805,297	569,410	86	52.38	3,275,987
FKII-L2-CM-DR1	Aspergillus calidoustus	*CaM*	99.5	MT704921	JAKLNK000000000	SRR18739382	Dichloran rose bengal agar	KSC-PHSF	313	39,874,905	569,221	154	51.11	6,174,864
FKII-L2-CM-PAB1	Aspergillus calidoustus	*CaM*	99.5	MT704923	JAKLNM000000000	SRR18739379	Potato dextrose agar	KSC-PHSF	371	40,055,042	622,573	131	51.11	5,891,335
FKII-L3-BK-PAB1	Aspergillus calidoustus	*CaM*	99.8	MT704928	JAKLNR000000000	SRR18739374	Potato dextrose agar	KSC-PHSF	385	40,340,333	676,118	123	51.19	7,181,415
FKII-L3-BK-DRAB4	Aspergillus calidoustus	*CaM*	99.8	MT704929	JAKLNS000000000	SRR18739373	Dichloran rose bengal agar	KSC-PHSF	301	40,773,058	742,271	149	51.02	8,633,370
FKI-L3-BK-DRAB1	Aspergillus calidoustus	*CaM*	99.8	MT704898	JAKLMT000000000	SRR18739398	Dichloran rose bengal agar	KSC-PHSF	429	40,391,628	505,550	186	51.06	10,486,873
FKII-L6-BK-DRAB1	Aspergillus tubingensis	*CaM*	99.6	MT704926	JAKLNP000000000	SRR18739376	Dichloran rose bengal agar	KSC-PHSF	88	35,008,407	662,607	168	49.13	5,784,450
FJII-L3-CM-P3	Aureobasidium pullulans	*ITS*	100.0	MT704887	JAKLML000000000	SRR18739407	Potato dextrose agar	JPL-SAF	258	28,497,861	345,423	45	50.30	8,272,240
FKI-L8-BK-P1	Exophiala oligosperma	*ITS*	99.5	MT704904	JAKLMV000000000	SRR18739396	Potato dextrose agar	KSC-PHSF	373	42,391,843	426,265	202	52.39	11,716,730
FJI-L2-BK-P3	Naganishia albida	*Tef1a*	96.5		JAKLLX000000000	SRR18739403	Potato dextrose agar	JPL-SAF	478	27,861,060	190,774	323	53.93	2,221,225
FJII-L4-SW-DR1	Penicillium biforme	*BenA*	98.6	MT704880	JAKLMG000000000	SRR18739412	Dichloran rose bengal agar	JPL-SAF	1,183	35,656,037	154,974	221	48.01	5,288,969
FJII-L5-SW-P3	Penicillium biforme	*BenA*	98.6	MT704892	JAKLMO000000000	SRR18739404	Potato dextrose agar	JPL-SAF	1,188	36,373,805	203,931	234	47.96	11,713,998
FKI-L3-CM-P1	Penicillium biforme	*BenA*	98.6	MT704911	JAKLNB000000000	SRR18739391	Potato dextrose agar	KSC-PHSF	824	35,881,231	221,716	136	47.78	5,808,819
FJII-L9-SW-P1	Penicillium brevicompactum	*BenA*	98.4	MT704893	JAKLMP000000000	SRR18739402	Potato dextrose agar	JPL-SAF	510	31,228,461	280,015	308	49.50	3,666,211
FJI-L10-BK-P2	Penicillium cerradense	*BenA*	94.5	MT704870	JAKLLZ000000000	SRR18739381	Potato dextrose agar	JPL-SAF	339	34,882,906	572,968	222	46.89	10,657,997
FJI-L3-BK-DG1	Penicillium cerradense	*BenA*	94.5	MT704871	JAKLMA000000000	SRR18739371	Dichloran glycerol agar	JPL-SAF	326	34,674,451	561,144	209	47.07	4,351,551
FJII-L3-CM-PAB4	Penicillium decumbens	*BenA*	97.8	MT704897	JAKLMS000000000	SRR18739399	Potato dextrose agar	JPL-SAF	216	24,008,368	508,196	327	50.43	4,033,252
FKII-L3-CM-DRAB1	Penicillium decumbens	*BenA*	99.2	MT704917	JAKLNH000000000	SRR18739385	Dichloran rose bengal agar	KSC-PHSF	281	26,798,112	500,337	217	50.02	6,316,652
FKII-L2-CM-P1	Talaromyces louisianensis	*BenA*	99.8	MT704919	JAKLNI000000000	SRR18739384	Potato dextrose agar	KSC-PHSF	364	35,494,196	689,550	114	46.07	4,017,393
FKII-L2-CM-DRAB3	Talaromyces louisianensis	*BenA*	99.8	MT704922	JAKLNL000000000	SRR18739380	Dichloran rose bengal agar	KSC-PHSF	385	35,499,968	735,697	108	46.07	6,621,100
FKI-L3-BK-DAB3	Talaromyces ruber	*BenA*	100.0	MT704905	JAKLMW000000000	SRR18739395	Dichloran rose bengal agar	KSC-PHSF	346	30,034,193	296,592	357	46.42	10,773,815
FJI-L7-BK-P1	Trichoderma rugosum	*Tef1a*	96.4	MT704867	JAKLLV000000000	SRR18739415	Potato dextrose agar	JPL-SAF	341	32,491,321	269,895	316	53.23	3,744,657
FJI-L10-BK-P1	Trichoderma rugosum	*Tef1a*	96.4	MT704875	JAKLMD000000000	SRR18739368	Potato dextrose agar	JPL-SAF	291	32,566,608	265,352	223	53.15	4,289,739
FJI-L10-BK-P3	Trichoderma rugosum	*Tef1a*	96.4	MT704876	JAKLME000000000	SRR18739367	Potato dextrose agar	JPL-SAF	240	32,648,028	288,145	238	53.06	3,683,568

a Identification was carried out as per recent literature and Houbraken et al. (10–15).

b KSC-PHSF, Kennedy Space Center-Payload Hazardous Servicing Facility; JPL-SAF, Jet Propulsion Laboratory-Spacecraft Assembly Facility.

In addition to radiation resistance and producing conidia that can withstand harsh environmental conditions, the fungi isolated in this study are also of clinical relevance (16–19). Alternaria alternata is highly UVC resistant compared to other common environmental fungi, such as Aspergillus and *Pencillium* species (20). The effects of UV on Aspergillus calidoustus are not yet reported; however, Aspergillus niger exhibits UV resistance (21). Similarly, members of *Exophiala* have high resistance to UVC, UVB, and environmental UV radiation (22). Aureobasidium pullulans belongs to a class of black yeasts that are resistant to UV and gamma irradiation (23, 24). Some strains of Naganishia albida are also resistant to UVA, UVB, and UVC (15).

UV resistance in the genus *Penicillium* varies, depending on the species and strain (25, 26). The members of *Talaromyces* are also resistant to UV radiation (27, 28). Some species of *Trichoderma* are resistant to UVC, and UV-inactivated spores show photoreactivation (29). The phenomenon of photoreactivation in such species is relevant to planetary protection and needs further attention.

### Data availability.

The WGSs and raw data have been deposited in GenBank under BioProject accession no. PRJNA800051. This project has also been deposited in the NASA GeneLab system (30) under project no. GLDS-497. The version described in this article is the first version.

## References

[1] Benardini JN, la Duc MT, Beaudet RA, Koukol R. 2014. Implementing planetary protection measures on the Mars Science Laboratory. Astrobiology 14:27–32. doi:10.1089/ast.2013.0989. 24432776

[2] Benardini JN, la Duc MT, Ballou D, Koukol R. 2014. Implementing planetary protection on the Atlas V fairing and ground systems used to launch the Mars Science Laboratory. Astrobiology 14:33–41. doi:10.1089/ast.2013.1011. 24432777

[3] Onofri S, de Vera JP, Zucconi L, Selbmann L, Scalzi G, Venkateswaran KJ, Rabbow E, de La Torre R, Horneck G. 2015. Survival of Antarctic cryptoendolithic fungi in simulated Martian conditions on board the International Space Station. Astrobiology 15:1052–1059. doi:10.1089/ast.2015.1324. 26684504

[4] Blachowicz A, Chiang AJ, Elsaesser A, Kalkum M, Ehrenfreund P, Stajich JE, Torok T, Wang CCC, Venkateswaran K. 2019. Proteomic and metabolomic characteristics of extremophilic fungi under simulated Mars conditions. Front Microbiol 10:1013. doi:10.3389/fmicb.2019.01013. 31156574 PMC6529585

[5] Checinska Sielaff A, Urbaniak C, Mohan GBM, Stepanov VG, Tran Q, Wood JM, Minich J, McDonald D, Mayer T, Knight R, Karouia F, Fox GE, Venkateswaran K. 2019. Characterization of the total and viable bacterial and fungal communities associated with the International Space Station surfaces. Microbiome 7:50. doi:10.1186/s40168-019-0666-x. 30955503 PMC6452512

[6] Be NA, Avila-Herrera A, Allen JE, Singh N, Checinska Sielaff A, Jaing C, Venkateswaran K. 2017. Whole metagenome profiles of particulates collected from the International Space Station. Microbiome 5:81. doi:10.1186/s40168-017-0292-4. 28716113 PMC5514531

[7] Chen S, Zhou Y, Chen Y, Gu J. 2018. fastp: an ultra-fast all-in-one FASTQ preprocessor. Bioinformatics 34:i884–i890. doi:10.1093/bioinformatics/bty560. 30423086 PMC6129281

[8] Bankevich A, Nurk S, Antipov D, Gurevich AA, Dvorkin M, Kulikov AS, Lesin VM, Nikolenko SI, Pham S, Prjibelski AD, Pyshkin A, Sirotkin Av, Vyahhi N, Tesler G, Alekseyev MA, Pevzner PA. 2012. SPAdes: a new genome assembly algorithm and its applications to single-cell sequencing. J Comput Biol 19:455–477. doi:10.1089/cmb.2012.0021. 22506599 PMC3342519

[9] Johnson M, Zaretskaya I, Raytselis Y, Merezhuk Y, McGinnis S, Madden TL. 2008. NCBI BLAST: a better web interface. Nucleic Acids Res 36:W5–W9. doi:10.1093/nar/gkn201. 18440982 PMC2447716

[10] Houbraken J, Verweij PE, Rijs AJMM, Borman AM, Samson RA. 2010. Identification of Paecilomyces variotii in clinical samples and settings. J Clin Microbiol 48:2754–2761. doi:10.1128/JCM.00764-10. 20519470 PMC2916617

[11] Cai F, Druzhinina IS. 2021. In honor of John Bissett: authoritative guidelines on molecular identification of Trichoderma. Fungal Divers 107:1–69. doi:10.1007/s13225-020-00464-4.

[12] Zheng L, Zhao J, Liang X, Zhan G, Jiang S, Kang Z. 2017. Identification of a novel Alternaria alternata strain able to hyperparasitize Puccinia striiformis f. sp. tritici, the causal agent of wheat stripe rust. Front Microbiol 8:71. doi:10.3389/fmicb.2017.00071. 28197134 PMC5281574

[13] Usuda D, Higashikawa T, Hotchi Y, Usami K, Shimozawa S, Tokunaga S, Osugi I, Katou R, Ito S, Yoshizawa T, Asako S, Mishima K, Kondo A, Mizuno K, Takami H, Komatsu T, Oba J, Nomura T, Sugita M. 2021. Exophiala dermatitidis. World J Clin Cases 9:7963–7972. doi:10.12998/wjcc.v9.i27.7963. 34621853 PMC8462220

[14] Houbraken J, Visagie CM, Frisvad JC. 2021. Recommendations to prevent taxonomic misidentification of genome-sequenced fungal strains. Microbiol Resour Announc 10:1128. doi:10.1128/MRA.01074-20. PMC8638587 34854710

[15] Bijlani S, Parker C, Singh NK, Sierra MA, Foox J, Wang CCC, Mason CE, Venkateswaran K. 2022. Genomic characterization of the Titan-like cell producing Naganishia tulchinskyi, the first novel eukaryote isolated from the International Space Station. J Fungi 8:165. doi:10.3390/jof8020165. PMC8875396 35205919

[16] Acland KM, Hay RJ, Groves R. 1998. Cutaneous infection with Alternaria alternata complicating immunosuppression: successful treatment with itraconazole. Br J Dermatol 138:354–356. doi:10.1046/j.1365-2133.1998.02091.x. 9602891

[17] Glampedakis E, Coste AT, Aruanno M, Bachmann D, Delarze E, Erard V, Lamoth F. 2018. Efficacy of antifungal monotherapies and combinations against Aspergillus calidoustus. Antimicrob Agents Chemother 62:e01137-18. doi:10.1128/AAC.01137-18. 30323034 PMC6256776

[18] Mittal J, Szymczak WA, Pirofski L, Galen BT. 2018. Fungemia caused by Aureobasidium pullulans in a patient with advanced AIDS: a case report and review of the medical literature. JMM Case Rep 5:e005144. doi:10.1099/jmmcr.0.005144. 29868175 PMC5982151

[19] Rimawi BH, Rimawi RH, Mirdamadi M, Steed LL, Marchell R, Sutton DA, Thompson EH, Wiederhold NP, Lindner JR, Boger MS. 2013. A case of Exophiala oligosperma successfully treated with voriconazole. Med Mycol Case Rep 2:144–147. doi:10.1016/j.mmcr.2013.08.003. 24432241 PMC3885957

[20] Valero A, Begum M, Leong SL, Hocking AD, Ramos AJ, Sanchis V, Marín S. 2007. Effect of germicidal UVC light on fungi isolated from grapes and raisins. Lett Appl Microbiol 45:238–243. doi:10.1111/j.1472-765X.2007.02175.x. 17718833

[21] Cortesão M, de Haas A, Unterbusch R, Fujimori A, Schütze T, Meyer V, Moeller R. 2020. Aspergillus niger spores are highly resistant to space radiation. Front Microbiol 11:560. doi:10.3389/fmicb.2020.00560. 32318041 PMC7146846

[22] Pulschen AA, Rodrigues F, Duarte RTD, Araujo GG, Santiago IF, Paulino-Lima IG, Rosa CA, Kato MJ, Pellizari VH, Galante D. 2015. UV-resistant yeasts isolated from a high-altitude volcanic area on the Atacama Desert as eukaryotic models for astrobiology. Microbiologyopen 4:574–588. doi:10.1002/mbo3.262. 26147800 PMC4554453

[23] Liu T, Zhu L, Zhang Z, Huang H, Zhang Z, Jiang L. 2017. Protective role of trehalose during radiation and heavy metal stress in Aureobasidium subglaciale F134. Sci Rep 7:17586. doi:10.1038/s41598-017-15489-0. 29242620 PMC5730648

[24] Cai S, Snyder AB. 2022. Thermoresistance in black yeasts is associated with halosensitivity and high pressure processing tolerance but not with UV tolerance or sanitizer tolerance. J Food Prot 85:203–212. doi:10.4315/JFP-21-314. 34614188

[25] Asthana A, Tuveson RW. 1992. Effects of UV and phototoxins on selected fungal pathogens of citrus. Int J Plant Sci 153:442–452. doi:10.1086/297050.

[26] Kawaguchi M, Kani A, Takatori K. 2019. Effects of UV irradiation on Penicillium strains isolated from a bread plant and the application to bakery products. Biocontrol Sci 24:179–183. doi:10.4265/bio.24.179. 31527350

[27] Sauceda-Gálvez JN, Roca-Couso R, Martinez-Garcia M, Hernández-Herrero MM, Gervilla R, Roig-Sagués AX. 2019. Inactivation of ascospores of Talaromyces macrosporus and Neosartorya spinosa by UV-C, UHPH and their combination in clarified apple juice. Food Control 98:120–125. doi:10.1016/j.foodcont.2018.11.002.

[28] Bhatnagar S, Aoyagi H. 2022. Thermal and UV degradation kinetics of water-soluble extracellular pigment produced by Talaromyces purpurogenus. Food Bioprocess Technol 15:606–619. doi:10.1007/s11947-021-02733-9.

[29] Sametz-Baron L, Berrocal T GM, Amit R, Herrera-Estrella A, Horwitz BA. 1997. Photoreactivation of UV-inactivated spores of Trichoderma harzianum. Photochem Photobiol 65:849–854. doi:10.1111/j.1751-1097.1997.tb01933.x.

[30] Ray S, Gebre S, Fogle H, Berrios DC, Tran PB, Galazka JM, Costes SV. 2019. GeneLab: omics database for spaceflight experiments. Bioinformatics 35:1753–1759. doi:10.1093/bioinformatics/bty884. 30329036

